# Occurrence and Quantification of Natural and Microplastic Items in Urban Streams: The Case of Mugnone Creek (Florence, Italy)

**DOI:** 10.3390/toxics10040159

**Published:** 2022-03-26

**Authors:** Valentina Rimondi, Alessio Monnanni, Eleonora De Beni, Gabriele Bicocchi, David Chelazzi, Alessandra Cincinelli, Sara Fratini, Tania Martellini, Guia Morelli, Stefania Venturi, Pierfranco Lattanzi, Pilario Costagliola

**Affiliations:** 1 Department of Earth Sciences, University of Florence, Via G. La Pira 4, 50121 Florence, Italy; alessio.monnanni@unifi.it (A.M.); gabriele.bicocchi@unifi.it (G.B.); stefania.venturi@unifi.it (S.V.); pilario.costagliola@unifi.it (P.C.); 2 IGG-CNR, Via G. La Pira 4, 50121 Florence, Italy; guia.morelli@igg.cnr.it (G.M.); pierfrancolattanzi@gmail.com (P.L.); 3 Department of Chemistry “Ugo Schiff”, University of Florence, Via della Lastruccia 3, 50019 Sesto Fiorentino, Italy; debenieleonora@gmail.com (E.D.B.); david.chelazzi@unifi.it (D.C.); alessandra.cincinelli@unifi.it (A.C.); tania.martellini@unifi.it (T.M.); 4 Consorzio Interuniversitario per lo Sviluppo dei Sistemi a Grande Interfase (CSGI), University of Florence, Via della Lastruccia 3, 50019 Sesto Fiorentino, Italy; 5 Department of Biology, University of Florence, Via Madonna del Piano 6, 50019 Sesto Fiorentino, Italy; sara.fratini@unifi.it

**Keywords:** microplastic, fibers, urban rivers, FTIR, Florence

## Abstract

The terrestrial environment is an important contributor of microplastics (MPs) to the oceans. Urban streams, strictly interwoven in the city network and to the MPs’ terrestrial source, have a relevant impact on the MP budget of large rivers and, in turn, marine areas. We investigated the fluxes (items/day) of MPs and natural fibers of Mugnone Creek, a small stream crossing the highly urbanized landscape of Florence (Italy) and ending in the Arno River (and eventually to the Tyrrhenian Sea). Measurements were done in dry and wet seasons for two years (2019–2020); stream sediments were also collected in 2019. The highest loads of anthropogenic particles were observed in the 2019 wet season (10^9^ items/day) at the creek outlet. The number of items in sediments increased from upstream (500 items/kg) to urban sites (1540 items/kg). Fibers were the dominant shape class; they were mostly cellulosic in composition. Among synthetic items, fragments of butadiene-styrene (SBR), indicative of tire wear, were observed. Domestic wastewater discharge and vehicular traffic are important sources of pollution for Mugnone Creek, especially during rain events. The study of small creeks is of pivotal importance to limit the availability of MPs in the environment.

## 1. Introduction

Urbanization produces the most dramatic physicochemical alterations to the fluvial ecosystems of the cities [1], resulting in what is known as the “urban stream syndrome” [2], which includes an increase in impervious surfaces, channel modifications and rectification, riparian vegetation removal, a decrease of biotic richness, and the degradation of water and sediment quality due to the input of contaminants [3,4]. In addition, the alteration of hydrology results in “flashier” flow regimes, which increase the risk and accentuate the side effects of flooding and drought, especially under future climate change scenarios [5].

In recent years, urban river rehabilitation, i.e., the return of degraded river ecosystems to a former pre-degradation ecological state [6], has been the focus of policies aimed at developing sustainable and resilient cities. Indeed, restoring rivers and creeks as wildlife corridors for multifaceted activities (urban parks, vegetable gardens, recreation facilities) favors social connectivity, increases the sustainability of water provision, provides mental and physical benefits to human health, and mitigates climate change effects [7,8].

Microplastics (MPs)—synthetic polymers of <5 mm in diameter [9,10,11], eventually bonding with additives, produced in micrometer size (primary sources) or derived from the weathering and degradation of larger plastic debris (secondary sources)—are abundantly present as contaminants in urban fluvial environments [12,13,14]. Microplastics pose a major threat to global aquatic ecosystems (e.g., [15,16,17,18]) as they cause severe impacts on the organisms after ingestion [19,20]. Moreover, MPs may act as both carriers and releasers of chemical pollutants adsorbed on MP fragments, posing a serious threat to river ecological functioning [15]. Despite the importance of deepening the knowledge on this topic, studies on the MP abundance in river catchments remain limited ([21] and reference therein), especially when compared to the marine environment. However, urban rivers, especially those crossing megacities, are considered the main source of land-based plastics to the seas [22], contributing 15–20% of the total global input to the oceans [15,23,24]. In the Mediterranean Sea, for example, a significant amount of MPs has been well documented at river mouths or in coastal lagoons [25,26,27,28].

Urban rivers receive MPs via atmospheric deposition, surface runoff (especially during storm events; [29]), industrial processes, and sewage treatment plants [30]. The occurrence of polymers, especially synthetic fibers, in sediments and stream waters is closely linked to anthropic activities, their abundance being directly related to population density and proximity to urban centers [31,32]. Microplastics can accumulate in the sediment column, the latter acting as a temporary sink [22]. Then, water flushing, and especially floods, inevitably transport the MPs into large rivers and, eventually, the open seas [33]. In general, MPs differ in appearance (fragments, fibers, and films), color, and composition (synthetic polymers, such as polyethylene PE, polyvinyl chloride PVC, and natural polymers).

Microfibers are fine strands used to make clothing, carpeting, and household items. Although overlooked in most studies or counted along with synthetic polymers [34], natural (e.g., cotton, yuta) or semi-natural (“regenerated”, nylon or rayon) fibers should also be distinguished or considered in environmental studies [35]. Although commonly perceived as eco-friendly due to their relatively quick degradation rate, natural microfibers are a global threat, comparable to synthetic polymers [35,36]. They account for the majority of anthropogenic litter found in ocean surface waters [34,37] and in marine animals, becoming vectors of organic and inorganic contaminants [35].

In this work, we characterize the distribution of synthetic and natural polymers in the sediments and waters of Mugnone Creek, which flows through the populated urban area of Florence (Italy), and quantify the fluxes of these contaminants in the stream waters. Mugnone Creek, a tributary of the Arno River, is a typical example of an urban stream suffering from extreme denaturation and poor ecological status [38,39], and its environmental requalification is one of the objectives of the city’s environmental policies. After discharging into the Arno River, the final fate of the found MP pollution is the Tyrrhenian Sea, a portion of the Mediterranean Sea. The latter, being a closed and densely populated basin, exhibits some of the highest concentrations of MPs and fibers in the world [27,34,40]. To the best of our knowledge, this work represents one of the first studies concerning synthetic and natural polymer pollution in Italian urban rivers, and it is an unprecedented eco-toxicological study on pollution in the urban area of Florence, a UNESCO world heritage site.

## 2. Materials and Methods

### 2.1. Study Area and Sampling

**Figure 1:**
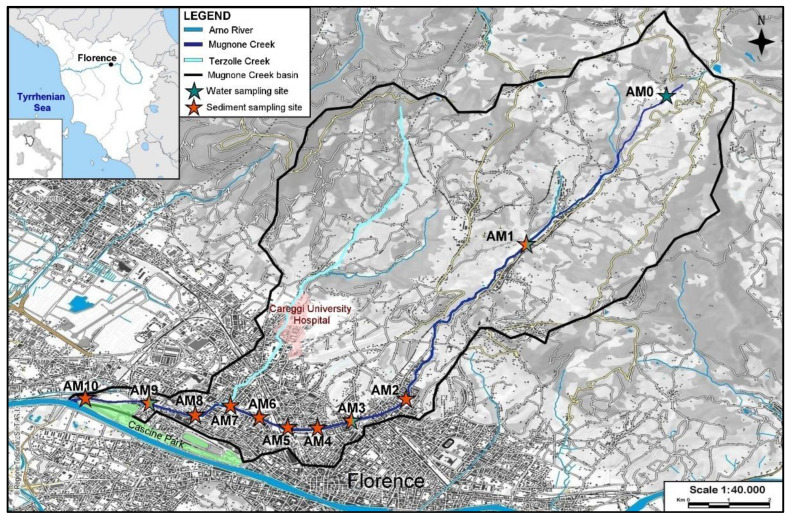
The urban area of Florence (Italy) and the location of sampling sites along the MC.

The urban area of Florence is one the most populated cities in central Italy (998,431 inhabitants on 1 January 2021). The city center is crossed by the Arno River. Among its tributaries, the 17 km long Mugnone Creek (hereafter MC) springs northeast of Florence and discharges at the western limit of the city in the Cascine park, i.e., the largest green area (ca. 130 ha) in Florence, after crossing a highly urbanized landscape. The MC drainage basin (60 km^2^) supports the urban pressure of 368,419 people (Florence district), with a high population density of 3601 inh/km^2^. During its path, it receives the waters from the Terzolle Creek (Figure 1), draining close to the University Hospital of Careggi. Since Roman times, the creek has been repeatedly diverted (in pre-Roman times, it discharged into the Arno River next to the famous Ponte Vecchio in the historic city center) in response to the needs of urbanization. Today, it is a water channel completely wedged in the urban fabric, with no possibility of natural evolution and with a high hydraulic risk due to the torrential regime [41]. Heavy autumn rains have recurrently caused flooding in the past, with extensive damage to surrounding neighborhoods. In addition, MC is the receiving water body of several sewer flood control tunnels dedicated to sewer overflow and stormwater management, directly discharging wastewater into the creek during intense rain events.

Sediments from MC were collected in June 2019 at 10 locations, moving from upstream of the Florence urban center (AM1–AM2) to the outlet into the Arno River (Figure 1). One sample (AM8) was collected just after the confluence with the Terzolle Creek to assess the impact of the hospital on the polymer pollution. Sediments were collected as grab samples with a metal scoop and stored in 1 L glass jars. In the laboratory, sediments were dried for two weeks at room temperature, covered with aluminum foil, and then sieved (metal sieve) at <2 mm to separate the finer fraction, which was investigated for MPs and natural fibers. Water sampling was repeated seasonally (June–September) for two years (2019–2020) at 3 of the 10 sediment collection sites (AM1, AM2, AM3). Upon considerations of the preliminary results from the 2019 sampling (see later), an additional site (AM0), further upstream of the Florence urban center (i.e., next to the MC spring), was sampled and characterized for micropolymers. River water (1–3 L) was collected 0–30 cm below the surface in triplicate using glass bottles pre-cleaned with acid, washed three times with stream water before sampling. Samples were stored in a dark place, at ~4 °C, until analysis. During water sampling, flow discharge was measured simultaneously at AM1, AM3, and AM9 by tracer dilution methods [42]. Daily fluxes (items/day) of synthetic and natural micropolymers at each site were calculated by knowing the number of items per cubic meter (items/m^3^) and the flow rate (m^3^/s) considered constant for 24 h at each site.

### 2.2. Microplastic Extraction from Sediment and Water

Extraction for the enumeration and identification of microplastic was performed by the density separation method ([43] and references therein) on a 50 g dry weight (hereafter d.w.) sediment subsample (<2 mm) from all sediment samples (AM1 through AM10). The sediment was then placed in a 500 mL glass beaker, shaken vigorously for 3 min with 300 mL of saturated NaCl solution, and then allowed to settle, covered with aluminum foil. After 24 h, the supernatant containing the plastic (or textile) items was filtered through a Büchner glass funnel using glass fiber filters (1.6 µm of pore size, Labchem), previously heated at 400 °C for 2 h. 

Because MP can commonly be found in table salt (e.g., [44]), the NaCl used for the density separation in this study comes from the Volterra salt deposit (southern Tuscany), dating back to the Messinian age (e.g., [45]), and is, thus, virtually MP-free. In addition, the saturated NaCl solution was filtered (0.45 µm) before its use to avoid any possible contamination, removing any impurities and MP fragments. For each sample, the extraction procedure was repeated three times using different glass filters to maximize the recovery of plastic items and to avoid filter-clogging or particle packaging from preventing numbering and identification. To overcome the problems associated with organic matter, which can mask the presence of polymers, all the three filters for each sample were digested with 10 mL of H_2_O_2_ (15 % *v*/*v*) for 48 h, stored on a glass Petri disk, dried in a desiccator, and stored until analysis. The H_2_O_2_ solution used for each filter digestion and a subsequent rinse with MilliQ^®^ water was then filtered, using the same equipment as for NaCl extraction, while the digested (residual) filters were stored and dried. A total of six filters (three digested with H_2_O_2_ and three not digested) were then retrieved for each sample and studied for both synthetic and natural polymeric particles, here referred to by the general term of microplastics (MP*). The abundance of MP* is reported as the number of MP* for one kilogram of dry sediment (MP*/kg d.w.). The use of a NaCl-saturated solution for MP extraction showed for artificial sand samples spiked with MP (PE, PP, PS, and PU) a mean MP recovery rate of 85 ± 3%, while for PVC and PET, the recovery was 67 ± 3%. These data are in good agreement with that reported by [43].

Concerning water samples, they were filtered within 1 week of collection with the same equipment used for sediments. Due to the high amount of particulate matter (likely of vegetal origin), the volume of filtered water varied between 250 and 2400 mL until the clogging of the filter occurred. After filtration, the filters were stored on a glass Petri disk, dried under a desiccator, and stored until analysis. The abundance of MP* (synthetic and/or natural polymers) is reported as the total number of items in the stream water volume (items/m^3^). 

**Figure 2:**
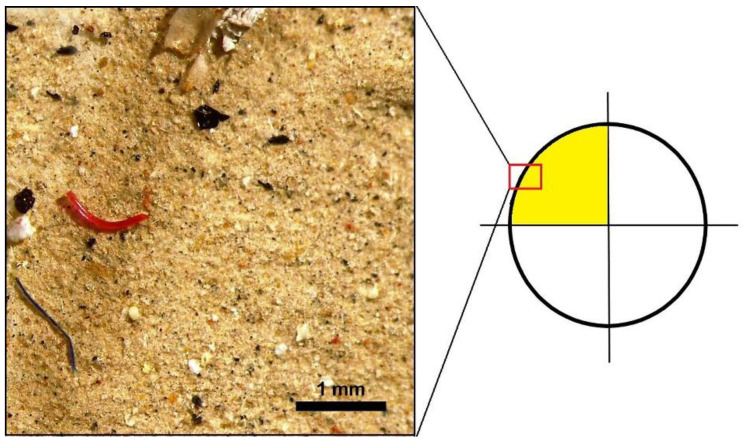
Example of MP* (red and black fibers) identified during binocular observations in the sediment sample and its position in the quarter of filter investigated (yellow area).

**Figure 3:**
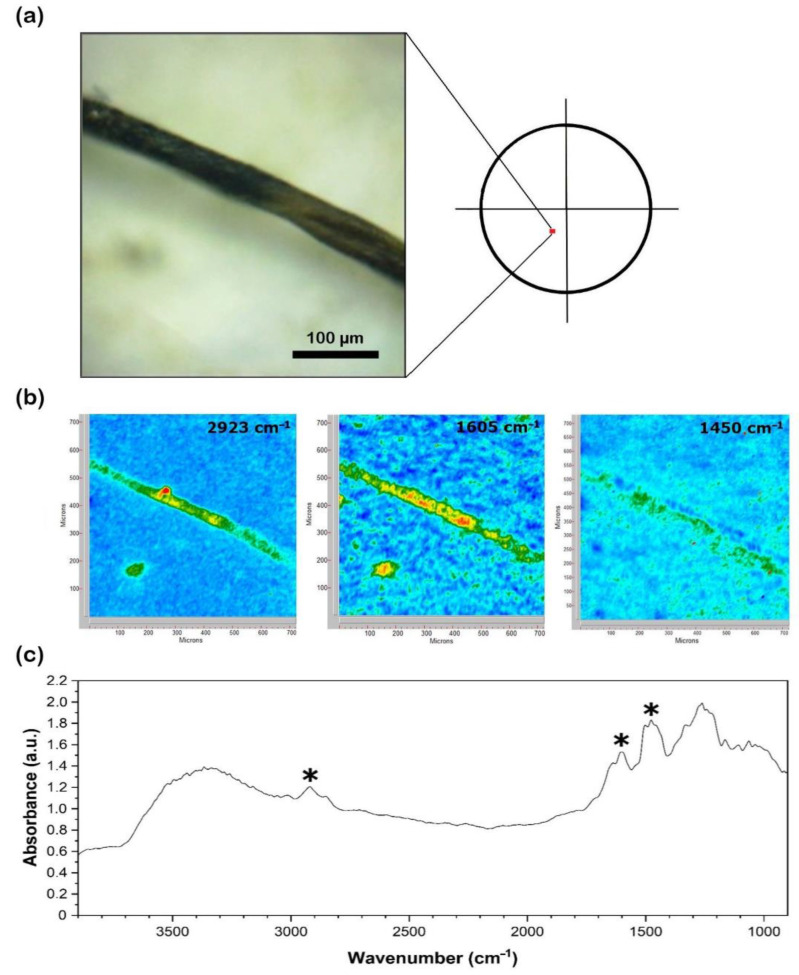
(**a**) Visible light map of the filter substrate with an SBR microfiber lying on it; (**b**) 2D FTIR imaging maps where the intensity of the following bands was mapped: 3000–2900 (aromatic and aliphatic CH stretch), 1605 (aromatic ring stretch), and 1450 cm^−1^ (CH_2_ bend). The chromatic scale of each map qualitatively shows the absorbance intensity as follows: blue, green, yellow, red; (**c**) FTIR reflectance spectra of the SBR microfiber and diagnostic bands (* symbol), assigned according to reference standards reported in [49].

**Figure 4:**
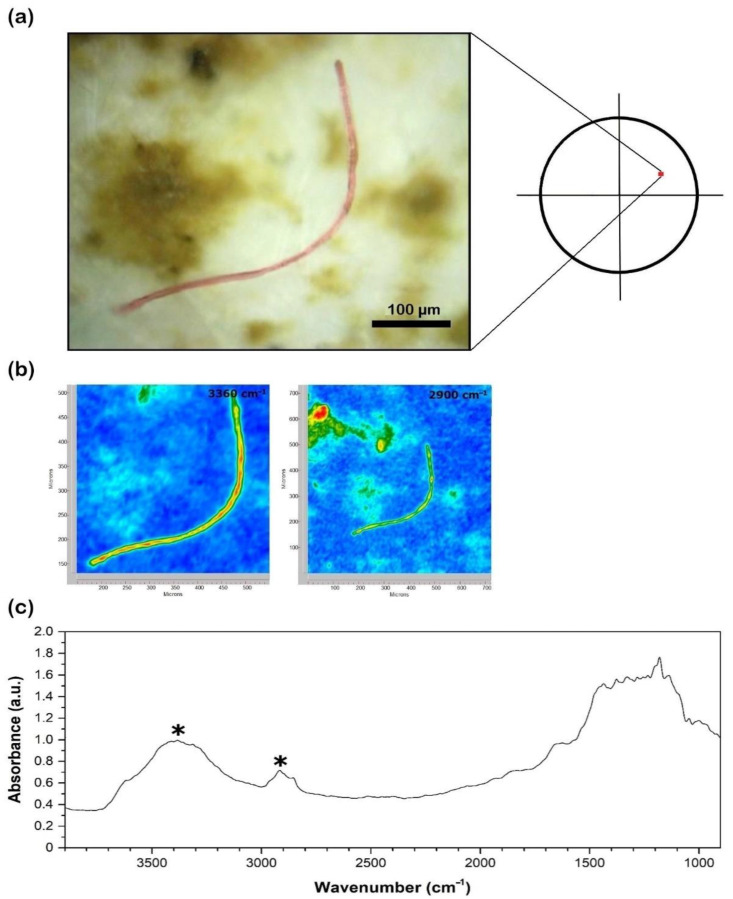
(**a**) Visible light map of the filter substrate with a cellulosic microfiber lying on it; (**b**) 2D FTIR imaging maps where the intensity of the following bands was mapped: 3500–3100 (O–H stretch, hydroxyl groups of the anhydroglucose unit), 3000–2900 (stretch of methyl and methylene C–H bonds). The chromatic scale of each map qualitatively shows the absorbance intensity, as follows: blue, green, yellow, red. Maps have dimensions of 700 × 700 μm^2^; (**c**) FTIR reflectance spectra of the cellulosic microfiber and diagnostic bands (* symbol), assigned according to reference standards reported in [50].

Binocular observations of the filters allowed for the count of particles in both sediments (Figure 2) and waters (Figure 3a and Figure 4a) and classification according to [46]. For sediments, only a quarter of each filter was observed (Figure 2) due to the extreme abundance of MP*, and the distribution of anthropogenic items was assumed to be homogenous. 

### 2.3. QA/QC Protocol

Microplastics are ubiquitous; thus, contamination can stem from air deposition on samples or equipment, the water used for cleaning equipment and sample processing, reagents, and the synthetic clothing worn by field staff. Thus, it is fundamental to check the potential contamination introduced to samples with background checks and field and procedural blanks. In this study, field blanks simulating every step of the collection procedure were performed. MilliQ^®^ water and a bottle blank were used to assess the cleanliness of the sample container. Laboratory blanks were performed to evaluate potential self- and cross-contamination in the laboratory and were included each time samples were processed and analyzed. The use of plastic sampling and laboratory equipment was eliminated wherever possible, and glass or metal was used in its place. Glassware was soaked with a concentrated detergent (Contrad 70 and Alconox) and rinsed three times with MilliQ^®^ water. The equipment was covered to reduce aerial deposition, and all analytical operations were conducted under a laminar flow cabinet located in a clean room. To minimize the sources of secondary contamination, personnel wore only cotton clothes and cotton lab coats and gloves in the laboratory space, even when not processing MP samples, and clothes were cleaned with a lint roller to capture any loose fibers. Baked glass fiber filters were put under the fume cupboard to check MP air deposition during filtering operations. Air blanks did not generally report MPs on them; however, in air blanks exposed during the processing of the water sampled in summer 2020, two blue fibers were observed. Therefore, these fibers were subtracted from the analyzed water samples.

### 2.4. 2D Imaging–Fourier Transform Infrared

The 2D imaging–Fourier transform infrared (FTIR) analysis of the plastic and non-plastic polymers in water samples (July 2019 and July–December 2020) was carried out on the dry glass fiber filters using a Cary 620–670 FTIR microscope, equipped with an FPA (Focal Plane Array) 128 × 128 detector (Agilent Technologies) and a Cassegrain 15× objective. This experimental setup was selected as it has proven to be highly effective in the identification of MP* fragments down to the micron size, even on complex matrixes rich with sediment [47]. Measurements were carried out in reflectance mode. Background spectra were collected on a gold plate surface. The glass fiber filters were analyzed directly, with no preparation steps required, and each analysis yielded a 2D “tile” map of 700 × 700 μm^2^ (128 × 128 pixels), where each pixel had a size of 5.5 × 5.5 μm^2^ and produced an independent spectrum (Figure 3b and Figure 4b). A background tile was acquired on the gold plate surface before each analysis. All the spectra (background and samples) were acquired in the 3900–900 cm^−1^ range, using 128 scans, an open aperture, and a spectral resolution of 8 cm^−1^. The detection limit of the detector to synthetic polymers (e.g., polyvinyl alcohol) was recently found to be as low as ca. 0.6 pg/pixel [48]. 

The pixel size of the FPA detector allows the collecting of a large number of independent spectra on the polymer microsamples; for instance, >150 independent spectra are typically acquired on a 1 mm long and 10 µm thick fiber in a single sample’s “tile” image (700 × 700 µm^2^). All the spectra were analyzed using Agilent Resolution Pro software (Agilent technologies). For each polymer, diagnostic bands were identified (Figure 3c and Figure 4c) and matched with those of references found in the literature [49,50]. In addition, the full spectral profile of each polymer was compared to the literature references to confirm the assignment. A selection of 2–5 diagnostic bands was imaged for each polymer: in the 2D FTIR maps, the intensity of characteristic bands of the investigated polymers was imaged with a chromatic scale of increasing absorbance, as follows: blue < green < yellow < red (Figure 3b and Figure 4b). Items were classified as synthetic (Figure 3) and natural (Figure 4) (cellulosic). In terms of dimensions, particles > 20 µm were characterized.

## 3. Results

### 3.1. Sediments

**Figure 5:**
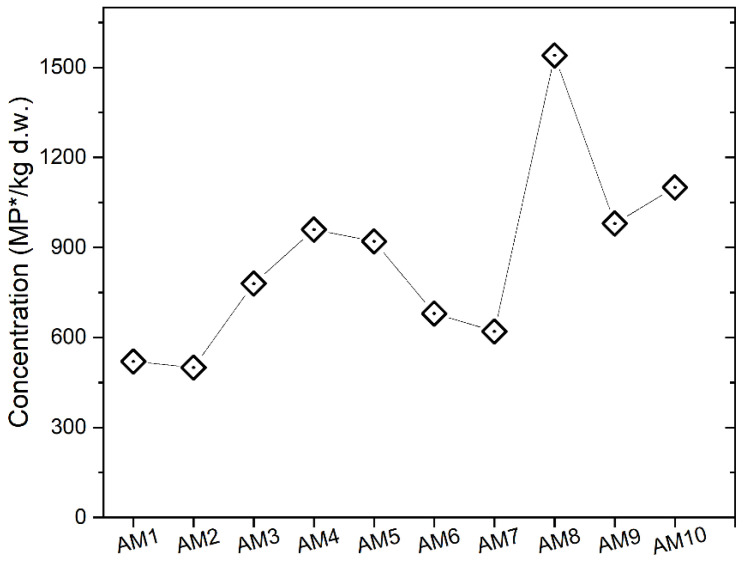
Contents of MP* (MP*/kg d.w.) in sediments of the MC at the AM1–AM10 sampling sites.

**Figure 6:**
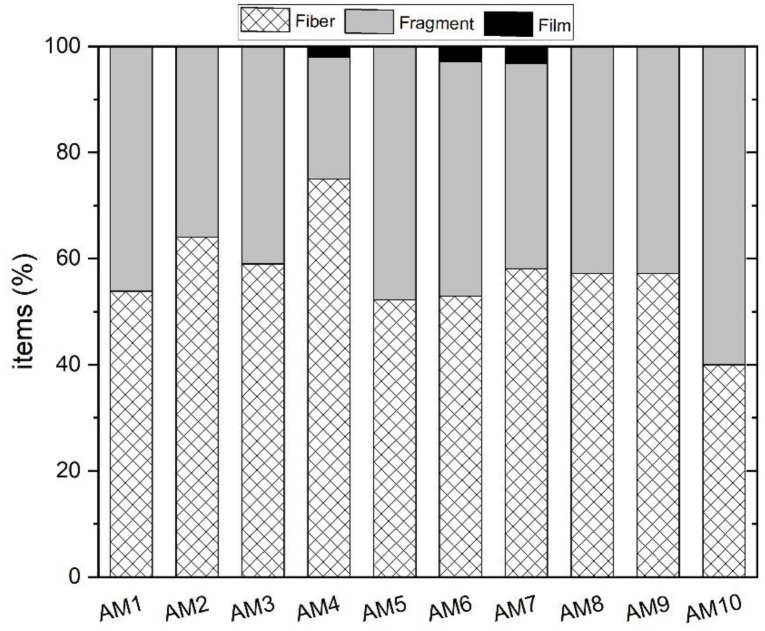
Fibers, fragments, and films (%) repartition of MP* in the MC sediments.

A total of 340 items were counted in the MC sediments through binocular observations, yielding MP* contents ranging from 500 to 1540 items/kg d.w. (Figure 5), with an average value of 860 ± 360 MP*/kg d.w. (1 SD). Fibers were the dominant shape class observed at all sampling sites (52–75%, mean 59 ± 7%), except at site AM10 (40%), corresponding to the outlet of MC into the Arno River (Figure 1), where fragments were most abundant (Figure 6). Films were poorly represented (<5%), while pellets were never observed. The lowest number of items (500–520 items/kg d.w.) was detected in AM1 and AM2, both located upstream of busy urban roads. Concordantly, a general increase in MP* was observed approaching urbanized areas and heavy-traffic roads, denoted by site AM3 (Ponte Rosso, Figure 5). The highest concentrations were reported for AM8 (1540 items/kg) and AM10 (1110 items/kg d.w.), corresponding to sediments collected after the confluence with the Terzolle Creek and at the MC outlet, respectively (Figure 1). 

### 3.2. Waters and Fluxes

**Figure 7:**
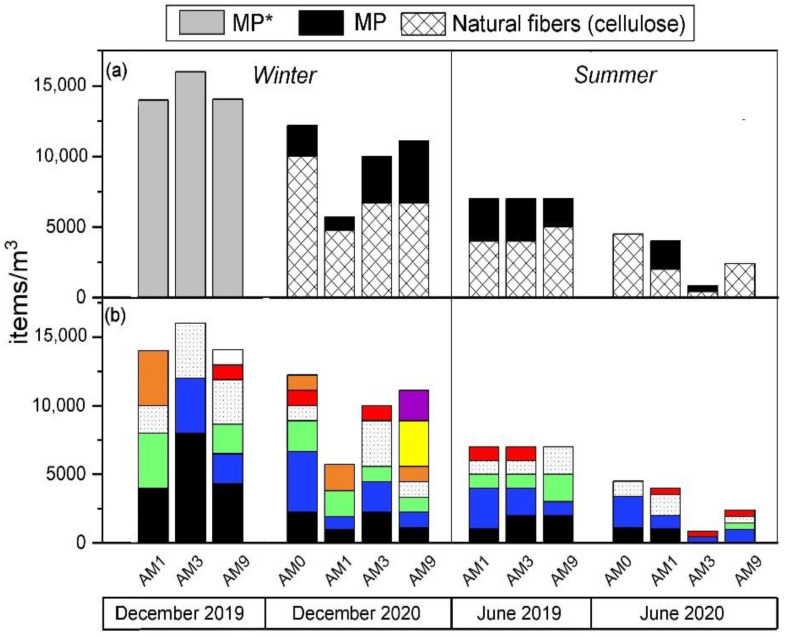
Items/m^3^ in the water of the MC at the four different sampling sites (AM0, AM1, AM3, AM9) and different sampling seasons. Classification based on (**a**) synthetic (MP) vs. natural (cellulosic) fibers and colors (**b**). In December 2019, no distinction between MPs and natural fibers was made. White dotted color refers to transparent particles.

**Figure 8:**
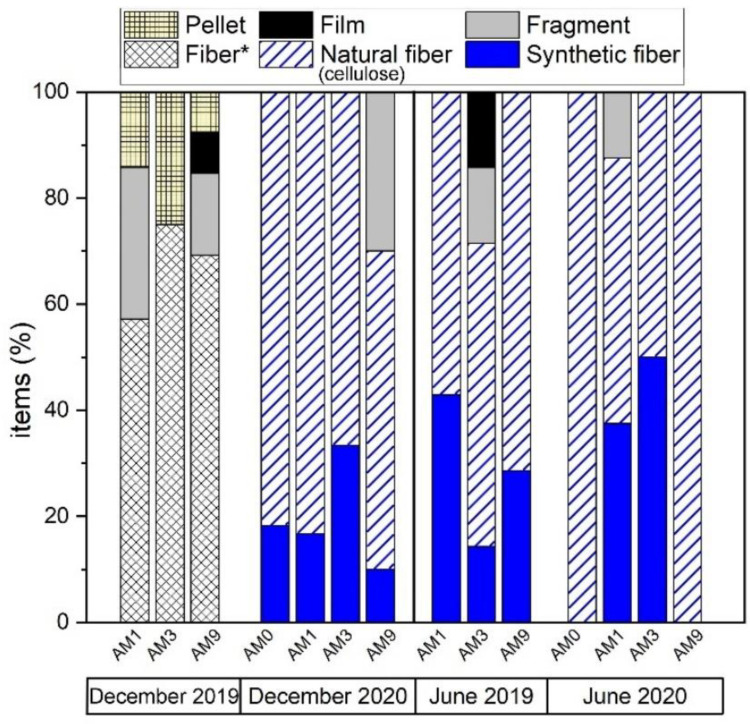
Particle classification (%) in water samples based on the shape. Fibers were distinguished as synthetic or natural or indicated with * when this distinction was not made.

Particle concentrations in MC waters ranged from 833 to 16,000 items/m^3^ (Figure 7). The lowest values were observed in June 2020 (833–4494 items/m^3^) and the highest in December 2019 (14,000–16,000 items/m^3^). Differences between sampling sites were quite modest, especially during the summer months. In terms of shape, fibers were the most abundant (57–100%) and the most representative for all seasons (winter and summer) at all sampling sites (Figure 8). At site AM3, fibers represented 100% of the particles during 2020 (Figure 8), and a peak of 12,000 fibers/m^3^ was found in the water during the 2019 winter sampling. Fragments accounted for 13–28% of the recovered items, while films and pellets were rarely observed (<15%). Particles were widely colored at all sites, especially during the winter months at AM9 (Figure 7b). Black and blue color were generally dominant. At AM3, up to 8000 black particles/m^3^ were observed during winter sampling in 2019. 

**Figure 9:**
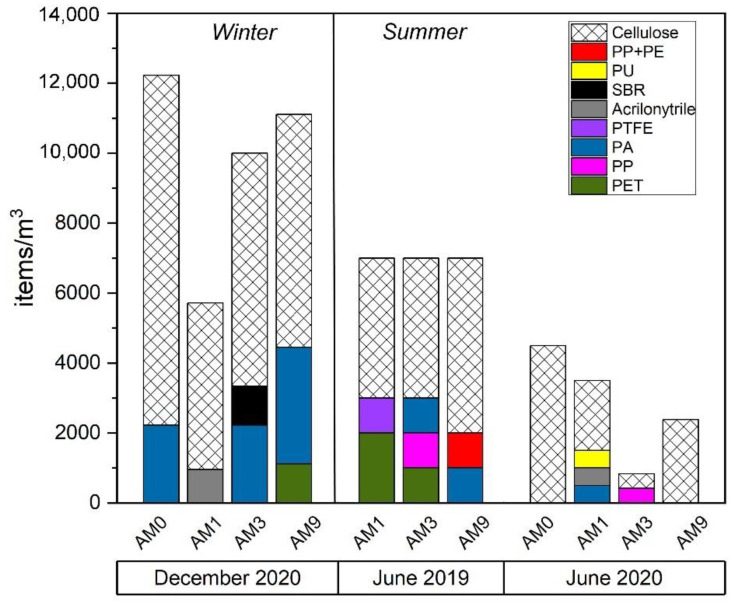
Classification of synthetic polymers in water samples. Natural polymers (cellulose) are also reported.

The FTIR investigation (*n* = 101) revealed the nature of the particles counted. Natural (cellulosic) fibers were 50–100% of the total at different sites (Figure 8), being equally distributed regardless of site location (i.e., upstream or downstream of the urban center). At site AM0, for example, the farthest upstream from the urban area of Florence, the highest number (10,000 items/m^3^) of cellulose-made particles was observed in December 2020. Polyamide (PA) was the most abundant polymer, observed in all seasons, especially during the winter sampling in December 2020 (Figure 9). It reached up to 3000 items/m^3^ at AM9 during this season, while the lowest values occurred in the summer months (500–1000 items/m^3^). Other polymers identified in MC were polyethylene terephthalate (PET), acrylonitrile, polypropylene (PP), a blend of PP and polyethylene (PE) (blend PP + PE), polytetrafluoroethylene (PTFE), butadiene-styrene rubber (SBR), and polyurethane (PU) (Figure 9). As a matter of example, the 2D FTIR maps of an SBR and cellulosic fibers are shown in Figure 3 and Figure 4, respectively. For the other class of polymers, representative visible and 2D FTIR maps are reported in the Supporting Information (Figures S1–S7). 

**Figure 10:**
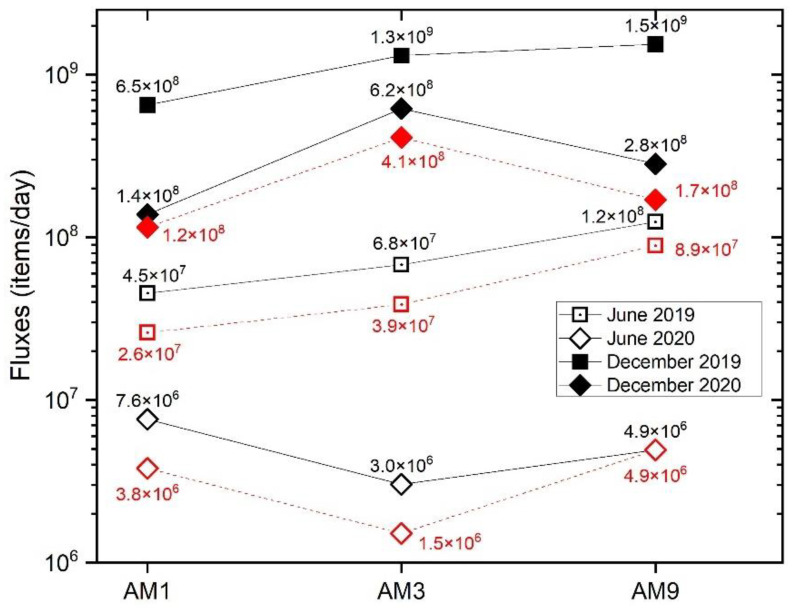
Fluxes (items/day) of total (MP*, black marker) and natural (red marker) particles at each sampling site along MC in the investigated seasons.

Water discharge measurements during sampling allowed us to calculate the fluxes of MP* (or natural) polymers at AM1, AM2, and AM3. Fluxes of total polymers (MP*) were in the order of 10^6^–10^7^ items/day during the dry months, while they increased up to 10^8^–10^9^ items/day during the wet seasons (Figure 10). In general, the fluxes of MP* at all sites were 1–2 orders of magnitude higher in 2019 than during the same season in 2020 (10^6^ against 10^8^ items/day). We observed that fluxes of cellulose material were of the same order of magnitude or even higher than those of MPs when polymer distinction was made.

## 4. Discussion

### 4.1. Spatial and Temporal Variations in Waters and Sediments

MC, which drains the highly urbanized area of the Florence district, is pervasively affected by MP* (synthetic and natural polymers) pollution in both water and sediment. Anthropogenic particle concentrations in water reached up to 16,000 items/m^3^ during the two-year sampling period of this study, significantly higher than those reported for other urban basins [12,51,52,53]. Surprisingly, the concentrations exceeded those observed for the Seine River (3–108 items/m^3^; [51]) and the Yangtze and Hanjiang rivers or some Chinese lakes (1660–8925 items/m^3^; [54]) by one to two orders of magnitude despite that these rivers flow through highly urbanized megacities (>10 million people). Different sampling methods could partly explain these large differences in MP* concentrations. Indeed, sample treatments may lack harmonized protocols for sampling and analysis, resulting in a misleading comparison of results, especially since the number of MPs in the water column is inversely related to the mesh size of the net used to collect them [13]. Most researchers applied a moderate mesh size (80–330 µm) for sampling to avoid net clogging (cf. [31,55]), thus missing the most conspicuous portion of MP* [56,57]. For example, [51] found that the abundance of small plastic fibers in an 80 µm net is one to two orders of magnitude higher than in a 330 µm net in the Seine River. Similarly, ~84% of plastic debris is in the 2–40 µm range for the Douro River in Portugal [57], and 90% of the total was less than 20 µm in the Elbe River [56]. The need to use a small mesh size during water sampling is genuine, particularly for microfibers, whose small widths allow them to easily pass through nets with mesh size > 63 µm [58]. 

To date, the few studies applying a <50 µm meshes for fluvial water sampling have reported a range of MP* similar or higher than that observed in MC. Hu et al. [59] estimated ~10,000 items/m^3^ in small rivers draining the residential areas of Shanghai (China), while 3097 to 9806 items/m^3^ were detected in urban sites in the Chinese Three Gorges Region [60]; in contrast, one to two orders of magnitude higher MP* were counted in the Saigon River draining the Ho Chi Minh megacity [61] and in the canals of Amsterdam (48–187 × 10^3^ items/m^3^; [62]).

The variability of MPs in sediments also relies on the methodology used. It has been shown that anthropogenic items with densities >1.2 g/cm^3^, such as PVC (ρ = 1.10–1.44 g/m^3^), may not be fully extracted from sediment fractions [43] using saturated NaCl solution (density of 1.2 g/cm^3^).

However, the separation of MPs with the 1.2 g/cm^3^ solution dominates in the scientific literature (e.g., [22]), providing more studies for comparison. Among the highest MPs in fluvial sediments, Hurley et al. [33] reported the case of the Mersey-Irwell catchments near Manchester (2.5 million people), with up to ~55,000 MP/kg (concentrations for <1.2 g/cm^3^ extracts). Besides this, the range of 500–1540 items/m^3^ for MC is generally higher than those observed for other Italian river systems ([28] and references therein) or abroad [60]. The spatial pattern well indicated an increase of contaminants as the creek enters the urban area (from site AM3 downstream), suggesting greater availability of anthropogenic debris in the basin. A peak of MP* (1540 items/kg) was associated with site AM8 (Figure 5), sampled a few meters downstream of the confluence with Terzolle Creek. This effluent drains the Careggi University Hospital, one of the largest in central Italy (about 1,000,000 hospitalizations each year), where plastic use in medical facilities is widespread, probably representing an additional source of trash for surface river networks.

### 4.2. Fluxes of Microplastics

Daily fluxes of MP* in MC were calculated for the four different sampling seasons at the three sites. They spanned over three orders of magnitude (10^6^–10^9^ items/day, with average values of 2.1 × 10^8^, 5.0 × 10^8^, and 4.9 × 10^8^ items/day at AM1, AM3, and AM9, respectively). The spatial pattern differed between the two years of monitoring, showing a moderate increase in MP* fluxes from upstream to downstream sites in 2019, especially in winter (from 6.5 × 10^8^ to 1.5 × 10^9^ MP* items/day from AM1 to AM3), while nearly constant values were observed in 2020 (Figure 10). This non-increasing pattern is not straightforward to explain, considering that potential MP* inputs are abundant between AM1 and AM9 sites, including the populated city of Florence and the Careggi University Hospital. This unexpected trend was also observed in the Seine River [53] and attributed to the non-conservative behavior of plastic polymers, which could sediment towards the riverbanks or streambed, as highlighted by the distribution in sediments between AM8 and AM9 (Figure 5), and/or migrate towards the aquifer that is locally fed by MC in the section between AM4 and AM5 and/or MC (e.g., [63,64]). In this sense, sediment monitoring has allowed us to depict more clearly the urbanization inputs in the Florence city area, although it cannot be excluded that contamination hotspots may also change rapidly in this matrix in response to fluvial dynamics [33]. 

Averaged anthropogenic particles fluxes per site were 1.2 × 10^9^ (2019) and 3.5 × 10^8^ (2020) items/day in December, and 7.9 × 10^7^ (2019) and 5.2 × 10^6^ (2020) items/day in June, being markedly higher during wet seasons. This effect is well-known in the literature [65,66] due to increased washing of impervious surfaces such as roofs and roads and to the scouring of sediments and re-suspension of particles due to the higher energy and more turbulent river flow that occurs during rain events. The MC has a torrential regime, and a short time frame (a few hours, depending on the kind of rain event and its intensity, e.g., [67]) is required to transport particles from the spring to the confluence with the Arno River. In December 2019 and 2020, samplings occurred after a couple of days of almost no precipitation (0.3–4 mm of cumulative precipitation in the 48 h before sampling), then these loads could be considered normal runoff conditions for MC during the winter months. The sampling of plastic debris at the crest of a flood stage is expected to greatly increase these fluxes [68] and could be of interest in establishing a realistic annual estimate of plastic debris entering the Arno River. 

As a final observation, our data consistently showed higher fluxes in 2019 than in 2020 (Figure 10). The latter year coincides with the spread of the coronavirus (SARS-CoV-2) and the explosive growth in the use of face masks (essentially made of synthetic polymers), which have been abundantly recovered as waste in rivers, beaches, and shorelines [69]. Although abandoned facemasks were also observed in the MC during the 2020 sampling campaigns, the creek waters did not show an increase in contamination. This could be due to multiple reasons, including: (i) the degradation rate of face masks in fluvial systems is currently unknown, so perhaps more aging is needed for degradation; (ii) national restrictions due to the SARS-CoV-2 pandemic, such as the lockdown (from 9 March to 4 May 2020 for all of Italy) of activities and public services, regulations prohibiting all movement by individuals except for justified reasons such as work, health, or other urgent needs, the halt of mass tourism, and the consequent decrease in road mobility reduced the availability of pollutants in the environment [70], comprising MP*, balancing the expected increase due to face-mask littering.

### 4.3. Polymer Shape and Type

Microfiber production has nearly doubled over the past 20 years, peaking at 111 million metric tons in 2019 [71]; the fibers are then the most representative anthropogenic particles found by environmental surveys in both oceans and fluvial settings [34,51,52,53,54]. MC was not an exception to this trend, and fibers dominated in both sediments and waters, sometimes representing the only item type recovered per sampling season (Figure 8). Fibers mostly originate from clothes washing, with fabrics releasing up to 10^7^ fibers/kg [72], which escape from wastewater treatment plants (WWTPs) and then enter the urban environment via wastewater effluents [73]. Atmospheric fallout [53] and/or road surface runoff can add a significant contribution to the system (see later). Among fibers, cellulosic fibers predominated (>50%) in MC (Figure 8), as generally testified for freshwaters [36] and for oceanic waters [34], seafloor sediments [74], ice cores [75], marine organisms [76], and, finally, human lungs [77]. As synthetic fibers occupy 2/3 of the global fiber production market [78], this mismatch between world production and current water column composition is an intriguing task of environmental research [34]. One plausible explanation is that cotton, rayon, and wool release more fibers than polyester (~52% of global synthetic fiber production; [78]) during laundering [79] or/and that the time required for natural fiber degradation is longer than expected due to end processing, such as the use of softeners, flame retardants and resins [34]. Despite the cause behind this longevity remaining unknown, the present study indicates that up to 10^8^ cellulosic fibers can be discharged daily into the Arno River in wet seasons (considering data from the AM9 site), suggesting that even small creeks, running through highly industrialized and populated areas, could greatly influence the balance of microfibers reaching large fluvial systems [80] as the Arno River and, hence, the Mediterranean Sea.

The most commonly detected synthetic polymers in Florence water samples were PA (25–65%), and PET (9–38%), followed by SBR (18–20%) and PP (13–20%). Polypropylene and PET are among the main polymer types found in freshwaters ecosystems [55]. They are mainly used for plastic and garbage bags and beverage containers. Therefore, they could be derived from the primary degradation of these improperly disposed materials in the urban environment. Polyamide could come from various forms of textiles, including clothes and carpets [29]. On the other hand, a specific traffic-related source can be speculated for SBR. This polymer was detected in the waters of site AM3, a crucial urban traffic junction in the city of Florence (Figure 1), suggesting that it could be related to tire abrasion occurring during car driving [81]. Indeed, butadiene–styrene rubber constitutes 60% of tires in combination with natural rubbers and various additives [82]. Tire wear is a potential conspicuous source of MPs in urban environments [22], and Panko et al. [83] documented that nearly all (>99%) of the particles generated remain on the ground, accumulate in road dust [84], and are subsequently washed away by rainfall. Kole et al. [85] estimated that 5–10% of oceanic plastic in the oceans comes from tire degradation.

## 5. Final Remarks

Physical and chemical degradation of small urban streams can negatively impact urban inhabitant life. Restoring these green corridors improves social connectivity, promotes citizen well-being, and is one of the main objectives of recent European urban policies. In this sense, characterizing MP pollution in urban areas is a necessary preliminary step to promote the full restoration of these ecosystems. 

Mugnone Creek, which runs through the densely populated city of Florence in central Italy and flows into the Arno River, is severely affected by “microplastics” (referring here to the sum of anthropogenic particles of both natural and synthetic origin) pollution. Anthropogenic polymers are mainly fibers in shape and cellulosic in composition in both sediment and water. Loads calculations and absolute concentration in sediment indicated that the urban area had a major impact on the budget of the fluvial system as a consequence of wastewater discharge from the domestic laundry, drainage from a major and important hospital of the city, and road dust runoff from congested city areas. A seasonal study of waters and fluxes calculations suggested that the availability of “microplastics” in Mugnone Creek increases during wet seasons, reaching 10^9^ items/day at the creek outlet due to the washing of the impervious surfaces of the city during rain events. In 2020, the spread of the SARS-CoV-2 pandemic reduced the “microplastics” loads in the basin, possibly due to the lockdown restrictions and the decrease of tourism and road mobility in the city. Items characterization by FTIR indicated that styrene–butadiene rubber, a major component of tires, is among the most prevalent polymers in surficial stream waters near congested urban sites. 

The study of small streams, which are common in the urban environment and strongly associated with terrestrial MP source areas, is of pivotal importance since they can show concentrations of MPs similar or higher than in large rivers and help depict the sources and sinks of these contaminants. The results provide the first baseline data of “microplastics” pollution in stream water in the urban area of Florence, and the fluxes will contribute to estimating the MP pollution budget into the Tyrrhenian Sea. Moreover, future studies of synthetic polymers in MC may indicate the outcome of recent urban mobility policies in Florence, which have resulted in the construction of a new tramway system to reduce private car use.

## Data Availability

Not applicable.

## Supplementary Materials

The following supporting information can be downloaded at: https://www.mdpi.com/article/10.3390/toxics10040159/s1, Figure S1: Acrylonitrile; Figure S2: Polyamide (PA); Figure S3: Polyethylene terephthalate (PET); Figure S4: Polyethylene-Polypropylene (PE-PP) blend; Figure S5: Polypropylene (PP); Figure S6: Polytetrafluoroethylene (PTFE); Figure S7: Polyurethane (PU).
