# Supplementary material for: Occurrence and Quantification of Natural and Microplastic Items in Urban Streams: The Case of Mugnone Creek (Florence, Italy)
Source: Toxics. 2022 Mar 26;10(4):159. doi: 10.3390/toxics10040159 (PMC9025813; doi:10.3390/toxics10040159)

toxics

MDPI

Supplementary Materials: Occurrence and Quantification of Natural and Microplastic Items in Urban Streams: The Case of Mugnone Creek (Florence, Italy)

Valentina Rimondi, Alessio Monnanni, Eleonora De Beni, Gabriele Bicocchi, David Chelazzi, Alessandra Cincinelli, Sara Fratini, Tania Martellini, Guia Morelli, Stefania Venturi, Pierfranco Lattanzi and Pilario Costagliola

2D Imaging-Fourier Transform Infrared analysis of plastic and non-plastic polymers: visible and 2D FTIR maps, and main absorption bands used for the identification of polymers

For each class of polymer, we report below representative visible and 2D FTIR maps, where the main diagnostic bands are imaged, and the assignments of the characteristic absorptions.

Figure S1: Acrylonitrile.

Intense absorption bands in the 3,000-2,800 cm^{-1} region (C-H stretch), ~2,240 (C≡N stretch), 1,739 (C=O stretch), 1,450 (δas CH_2), and 1,360 cm^{-1} (δs CH_3).

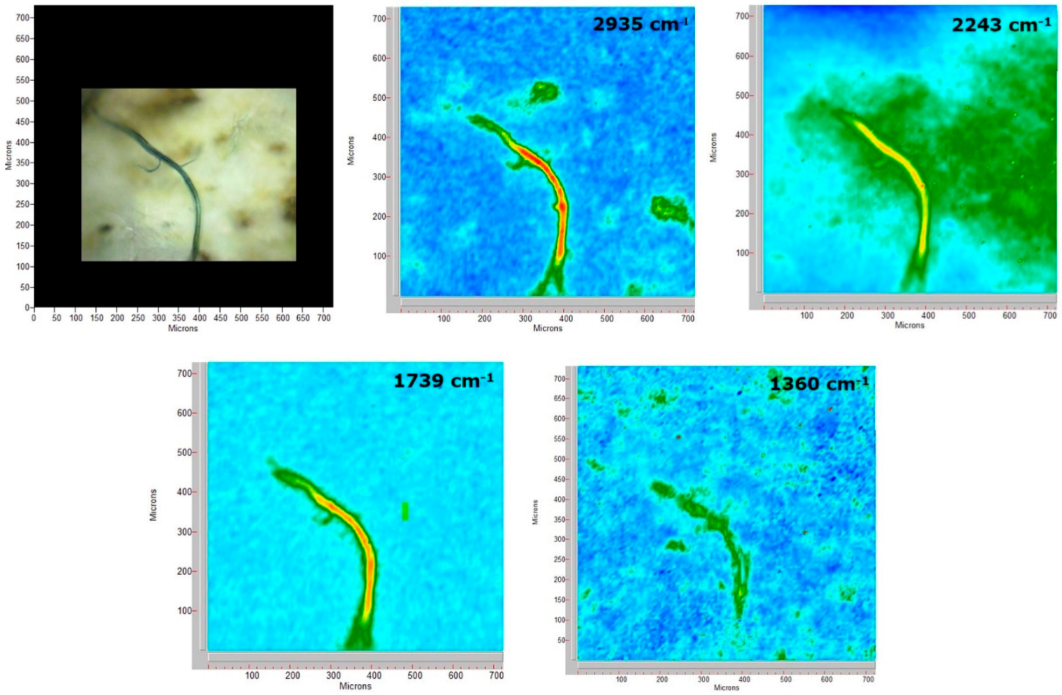

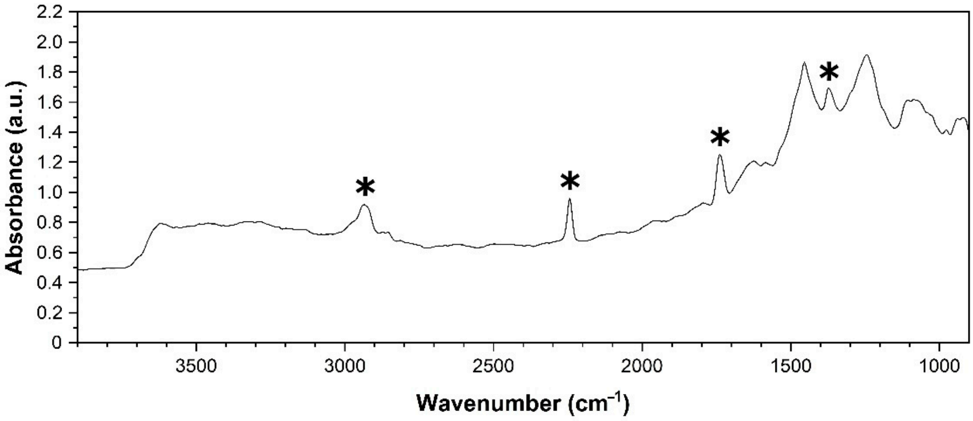

Figure S2: Polyamide (PA).

Absorptions in the 3,400-3,300 cm^{-1} region (N-H stretch), 3,000-2,800 (C-H and CH_2 stretch), 1,650 (amide I), 1,570 (C(O)-N-H bend, C-N stretch), ~1,450 cm^{-1} (CH_2 bend).

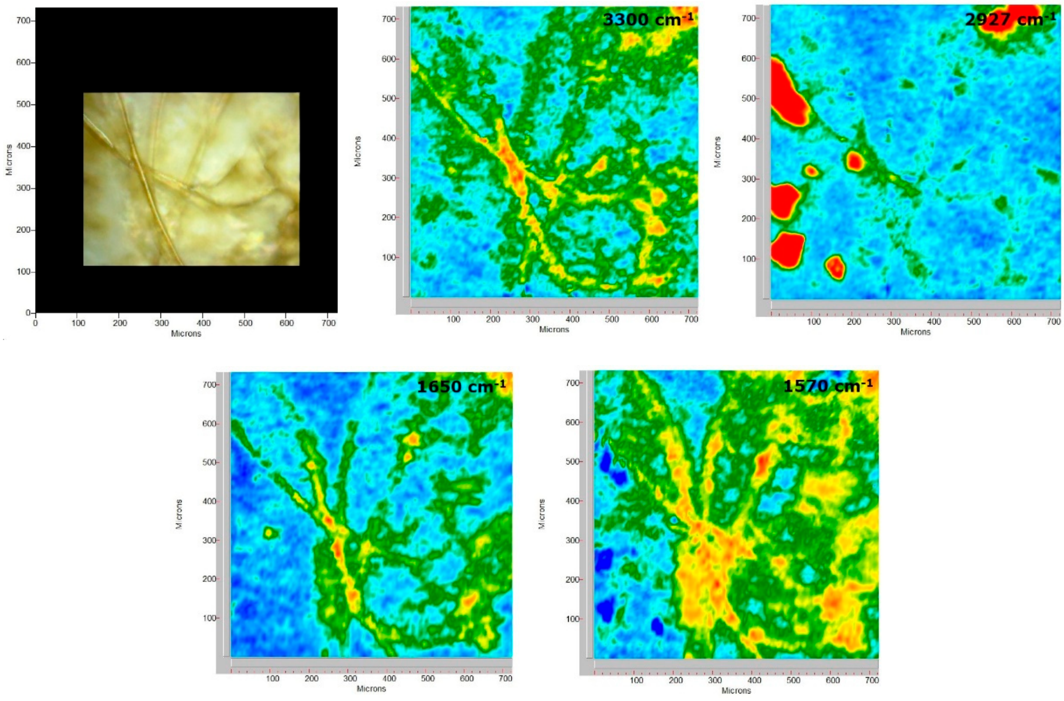

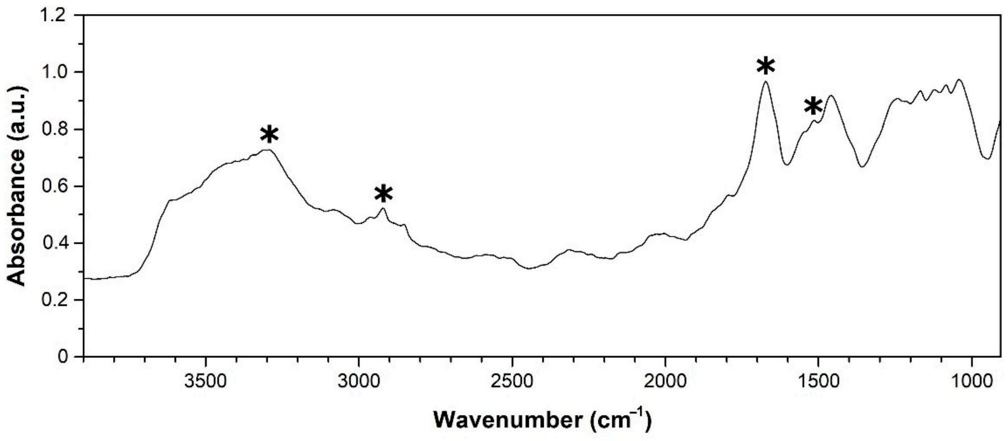

Figure S3: Polyethylene terephthalate (PET).

Bands at 3,000-2,860 cm-1 (aromatic and aliphatic CH stretch region), 1,730 cm-1 (C=O stretch), ~1,577 and 1,510 cm-1 (aromatic C=C stretch), 1,410 (aromatic skeleton stretch) and ~1,100 cm-1 (C-O stretch).

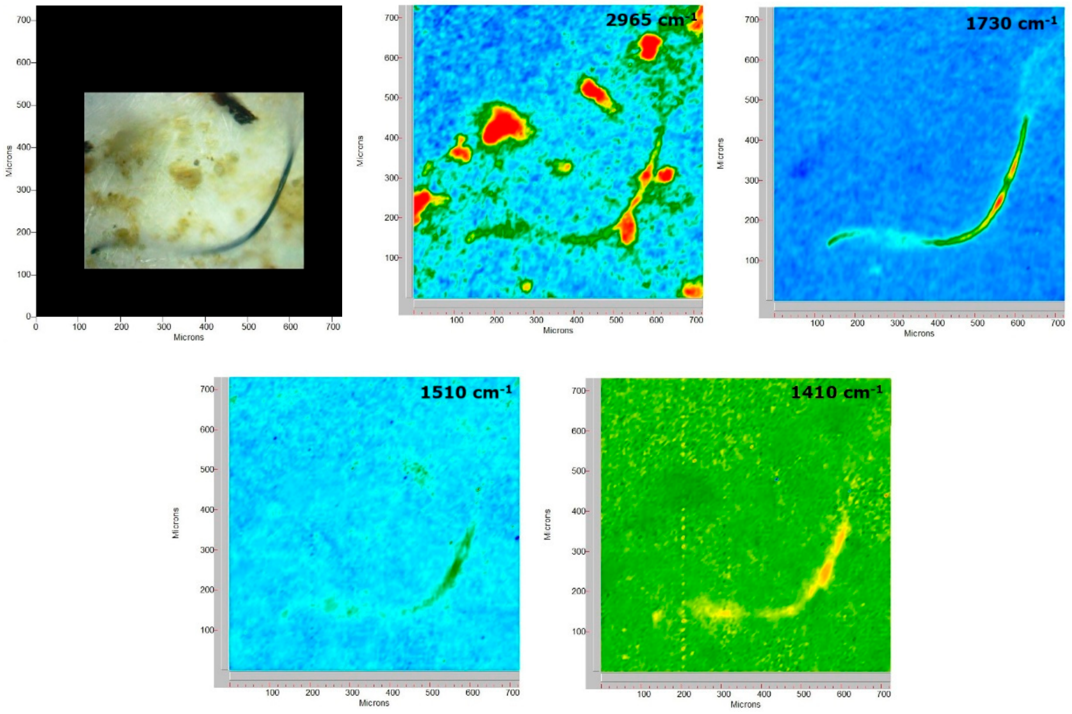

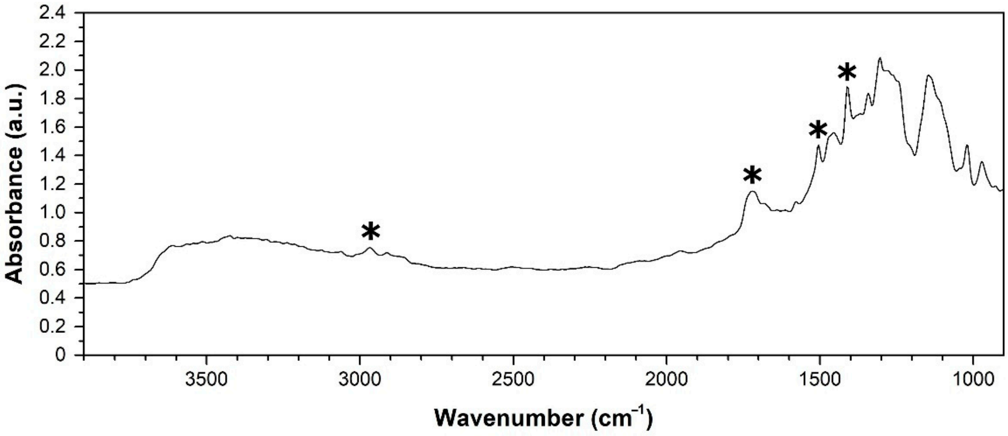

Figure S4: PE and PE-PP blend

PE is identified by absorption peaks at 2,920 and 2,850 cm^{-1} (CH stretch region), and ~1,465 (δ CH_2) cm^{-1}. PP is identified by absorption peaks at 2,950, 2,915 and 2,856 cm^{-1} (CH stretch region), at 1,458 (CH_2 bend), 1,373 (CH_3 bend) and 1,161 cm^{-1} (CH bend, CH_3 rock, CC stretch). Based on these assignments, the spectra of the fiber and fragment in the two sets of figures below might be assigned to, respectively, a PE-PP blend and PP.

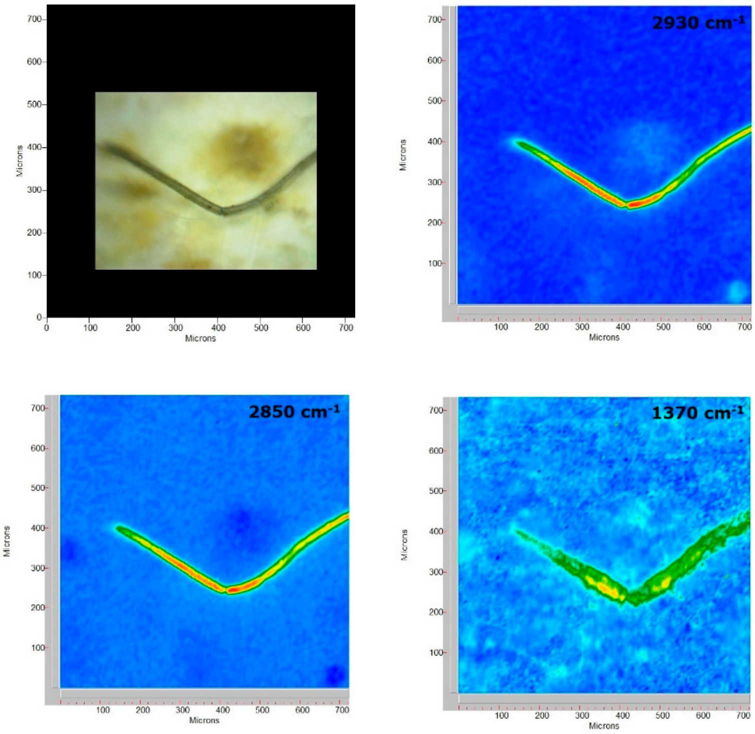

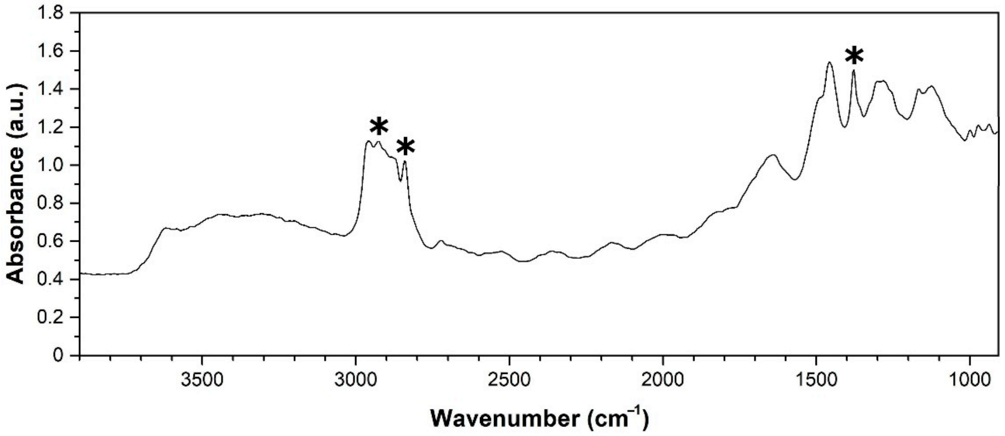

Figure S5: Polypropylene (PP).

Absorption peaks at 2,950, 2,915 and 2,856 cm^{-1} (CH stretch region), at 1,458 (CH_2 bend), 1,373 (CH_3 bend) and 1,161 cm^{-1} (CH bend, CH_3 rock, CC stretch).

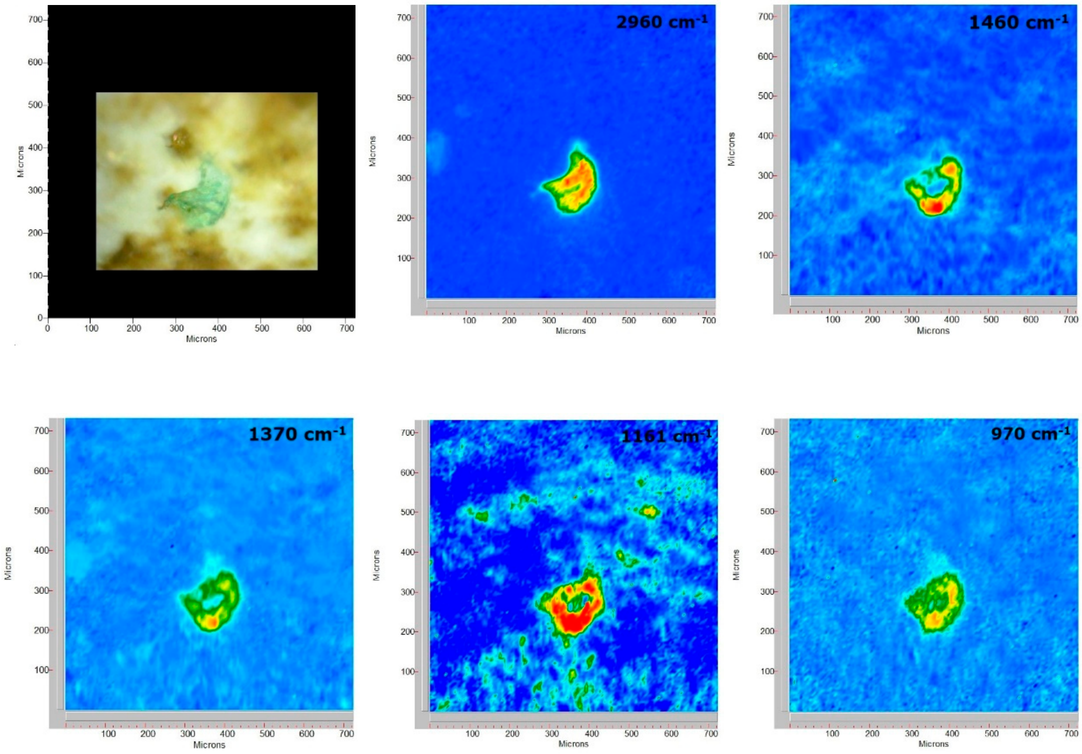

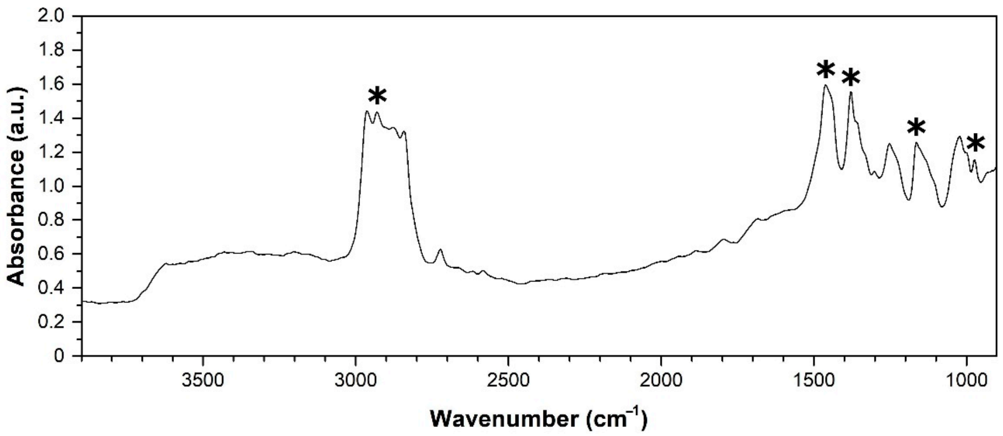

Figure S6: Polytetrafluoroethylene (PTFE).

PTFE is typically identified by two distinct peaks at ~1,200 and 1,150 cm^{-1}, assigned to the main peaks of polytetrafluoroethylene (PTFE), i.e. CF_2 symmetric stretch bands. In our case, the peaks appear to be shifted to higher wavenumbers, and there is some overlap with the bands of the underlying glass fiber filter. However, we can still tentatively identify the polymer as PTFE based on the presence of the two strong absorptions, and also considering the lack of CH (see map at 2,940 cm^{-1}, where no significant absorption from the fiber is observable as compared to the background filter absorption) and C=O stretching bands that rule out other polymer classes.

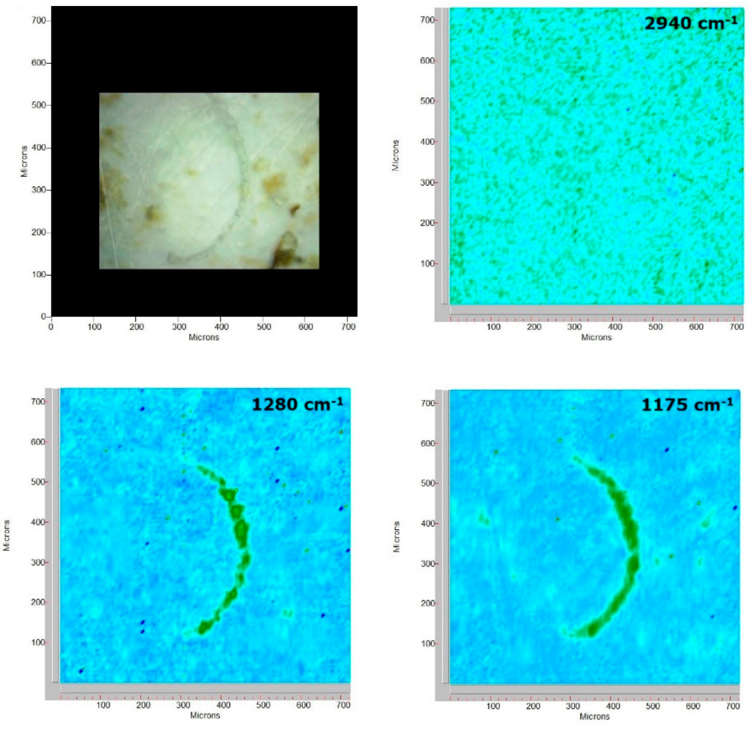

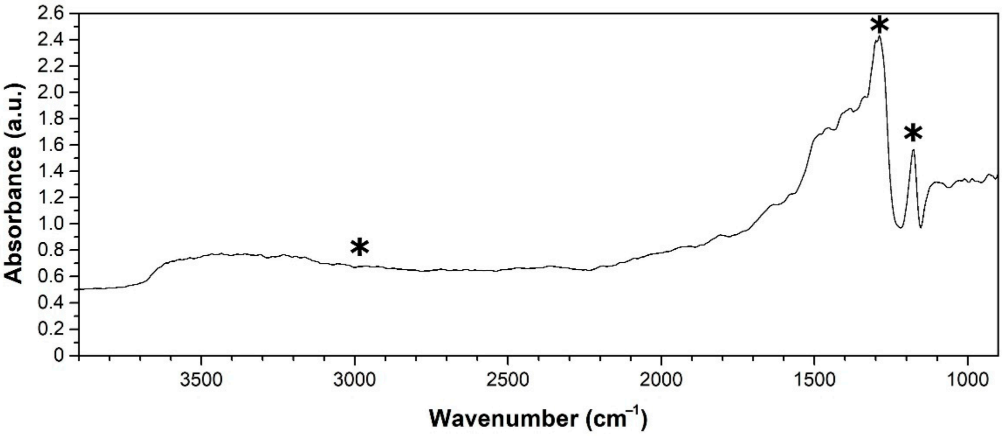

Figure S7: Polyurethane (PU).

PU is typically identified by peaks at ~1,730 (C=O stretch), 1,530 (C-N stretch), 1,450 (CH_2 bend) and 1,225 cm^{-1} (C(=O)O). In our case, the C=O stretch band was very pronounced, while that at 1,530 was less intense than what expected, which might be ascribed to a PU-acrylate composite. Overall, we assigned the fiber tentatively to a PU-based polymeric material.

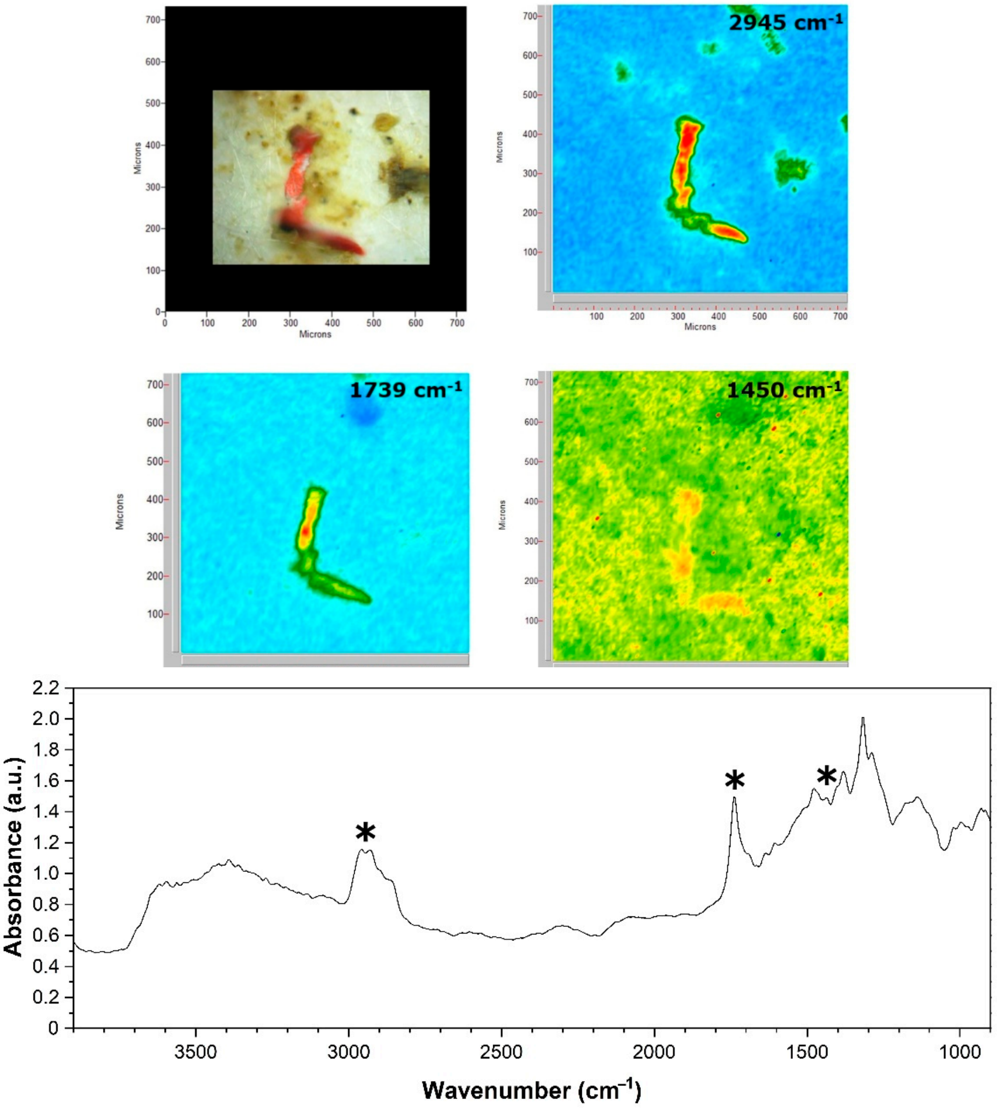

Supplement: Supplementary file 1 [file toxics-10-00159-s001.zip › toxics-1596006-supplementary.pdf]
